# Addressing the Unique Needs of LGBTQ+ Older Adults Living with Cognitive Impairment

**DOI:** 10.1002/alz70858_102457

**Published:** 2025-12-26

**Authors:** Sarah McCrackin, Nadine Austin, Jessica Vanderwerf, Nik M. Lampe

**Affiliations:** ^1^ University of South Florida, Tampa, FL, USA; ^2^ Georgia State University, Atlanta, GA, USA

## Abstract

**Background:**

Lesbian, gay, bisexual, transgender, and queer (LGBTQ+) older adults face substantial psychosocial barriers when seeking care services, support, and resources needed for maintaining their brain health. Studies suggest that LGBTQ+ individuals may face a higher risk for adverse brain health outcomes than their heterosexual, cisgender peers, underscoring the urgency of addressing these disparities. This study aimed to identify the unique healthcare needs of LGBTQ+ older adults living with cognitive impairment in the United States.

**Method:**

Between June 2024 and January 2025, the study team conducted semi‐structured interviews with community‐dwelling LGBTQ+ adults aged 60+ who were diagnosed with mild cognitive impairment (MCI) by a licensed clinician (*n* = 7); community‐dwelling LGBTQ+ adults aged 60+ who self‐reported significant cognitive problems (*n* = 8); and licensed clinicians who routinely provide care to LGBTQ+ older adults with cognitive impairment (*n* = 5). For study participation, LGBTQ+ community members without an MCI diagnosis must score below 19 on the MoCA BLIND English (Version 8.1). Data were coded by the study team using NVivo qualitative data management software and analyzed inductively through grounded theory analysis.

**Result:**

Preliminary findings underscore the critical need to identify and address the healthcare needs of LGBTQ+ older adults living with cognitive impairment, emphasizing the importance of fostering equitable memory care for LGBTQ+ people as they age. Three thematic themes emerged in the data: (1) the need for available and high‐quality social support; (2) the need for age‐friendly and LGBTQ+ culturally responsive resources; and (3) the need to reduce stigma when promoting greater resource utilization.

**Conclusion:**

Preliminary findings highlight the unique needs, experiences, and challenges faced by LGBTQ+ older adults living with cognitive impairment in the United States. Among the key themes, lack of social support emerges as a major concern, with the majority of LGBTQ+ older adult and clinician respondents emphasizing its structural impact on this patient population. The next steps for this study include assembling and collaborating with a Community Advisory Board to develop an evidence‐based program or set of strategies aimed at meeting the needs of LGBTQ+ older adults living with cognitive impairment.

